# A comparative study on marma and acupoints

**DOI:** 10.1016/j.jaim.2023.100769

**Published:** 2023-07-25

**Authors:** Tong Wu, Xing-yi Wang

**Affiliations:** Institute of Science, Technology and Humanities, Shanghai University of Traditional Chinese Medicine, Cailun Road 1200, Pudong New Area, Shanghai, China

**Keywords:** *Marma*, Acupoint, Ayurveda, Traditional Chinese medicine, Body view

## Abstract

*Marma* is an important component of Ayurveda. It was recorded in *Susruta Samhita* that there were 107 *marma* points in human body, which located at the anatomical site where muscles, veins, ligaments, bones and joints meet together and were regarded as seats of life energy. While acupoints in Traditional Chinese Medicine is defined as having the similar position as *marma* points, and also the function of regulating vital energy, these body points have been constantly combined with acupuncture technique, which encouraged doctors to stimulate applicable acupoints in order to relief pain and treat diseases. Given the similarity of their location and regulation of vital energy, this paper further conducted a systematic comparison of their origins, general features and clinical applications based on literature of Ayurveda and Traditional Chinese Medicine. The results indicated that the main difference existed in their origin, with *marma* from the battlefield culture of ancient India and acupoints from medical experience of ancient Chinese. In general features, they showed diverse characteristics of medical theories through classified methods. As for clinical applications, acupoints have always been closely related to medical science in the whole progression, yet *marma* points were gradually endowed with medical functions. Overall, these body points appeared independently and conveyed the different body views bred by two civilizations. Through cross-region comparison, people's understanding of each traditional medicine and cultural connotation behind it could be deepened, which assisted to achieve the cooperation and innovation of traditional medicine.

## 1 Introduction

Theories of traditional medicine are always constructed by ancients' cognition of the universe and human body as well as their general life experience. In the process of building and developing medical system, various regions and periods creating a cultural relation or gap, sometimes led to the same or different paths respectively. Taking individual traditional medicine in India and China into account, scholars might have found some specific signs. Inchoate writings of Ayurvedic medicine had contained detailed descriptions of *marma* points, which were referred to as acupoints of India by the Chinese scholar Liao Yuqun in his work concerning Ayurveda [1]. Ayurveda classical texts like *Susruta Samhita* (here in after called '*S. samhita*')and *Astanga hrdayam* described 107 *marma* points in human body, and regarded them as fatal spots or vital points, due to their anatomical sites where muscles, veins, ligaments, bones and joints meet together and their natural feature as seats of vital life force [2,3]. Knowledge of these 107 points was honored as half of science of surgery, and on the basis of them appeared *marma* therapy, which gradually undertook the functions of treating diseases. Undoubtedly, there existed similarities between *marma* points and acupoints of Traditional Chinese Medicine (TCM). In the perspective of TCM, acupoints have specific morphological characteristics like *gu kong* (bone hollow) and *mai dong* (stirred pulse) [4], along with abstract definition of “the locations where the spirit *qi* pass, where they exit and enter” [5]. As for acupoints therapy, it was originally and completely bound up with acupuncture, which constituted the most essential component of external treatment in TCM. With similar medical descriptions and applications of *marma* and acupoints, speculations were sparked by researchers about their medical source, especially when *marma* therapy was regarded as a part of *Suchi Veda*, whose long history was no less than TCM. Such scenarios confused people about the origin of acupuncture [6,7]. In this paper, we collected medical descriptions of both sides by combing important literature of Ayurveda and TCM as well as historical backgrounds, and conducted a systematic comparative study with the aim of clarifying the source and deepening people's cognition of body views in different civilizations.

## 2 The origin

From the perspective of etymology, the word *marma* is derived from the root *mri* with suffix *manin*, meaning seat of life or meeting place in Sanskrit. English translators regard it as a kind of fatal spot, vital point and vulnerable point, which all implies the structural characteristics of *marma* and its apparent importance over other parts in the body.

Knowledge of *marma* dated back to the Vedic era, when there happened continuous wars in ancient India. From earlier classical Indian scriptures including the Vedas, we could generally understand the original meaning of *marma*. As *Rigveda*, the book recording Vedic hymns or sacrificial formulas, once stated, “for battle, with which he found the mortal spot of that very Vrtra, as, gaining mastery, he thrust with the thrusting mace, while conferring who knows how much” [8], and the verse like “your vulnerable places I cover with armor; let Soma the king clothe you with immortality” [8], it is obviously during the war that the soldiers found, if they attacked the enemies' *marma* points, the enemies would be more likely to fall down, lose ability of movement or even die. In view of this, the soldiers were required to both put on armors to defend their own *marma* points and vigorously attack the enemies’. Besides the armor, prayers and hymns can also be served as protection. In hymns of *Atharvaveda*, the ancients earnestly prayed, “O *prana*, to thy lightning, reverence, O *prana*, to thy rain! when *prana* calls aloud to the plants with his thunder, they are fecundated, they conceive, and then are produced abundant plants” [9]. It spoke of *prana*, which means life or breath in Sanskrit, and *marma* is exactly considered as the seat of *prana*, so damage here will result in a loss of vital life energy. However, more details could not be attained in Vedas, even in its branch *Upanishads* or *Puranas*, only some locations were sporadically described. It was not until *S. samhita*, one of three Ayurvedic classics came out, that systematic knowledges of *marma* were clear. This book opened a separate chapter for *marma*, standardized the locations of 107 *marma* points in human body and figured out their different classifications [2]. More importantly, it recorded each anatomical detail of these points, reflecting the former cognitive level of human body. Thus, the medical connotation of *marma* was established and became the consensus of Ayurvedic texts thereafter.

The above discussed the origin, that is to say, it is actually an extension of medical knowledge from battlefield culture. But when talking about the system of acupoints in Chinese medicine, academic community generally believed that acupoints originated from ancients' long-term living experience which contained progressive discovery and accumulation in medical practice [10,11]. In old times, people found that pressing or touching some certain body regions by hands can relieve the pain of the body. Slowly, they attempted to apply tools like stone needles, medal needles instead of hands on these regions for better pain-relieving effects. As time went by, people realized the particularity of these positions and the connection between them. However, this instinctive behavior had not risen to rational behaviors, unable to prove the discovery of acupoints, and it was only after a long period of medical experience that the concept of acupoints was eventually formed in the period of *Huangdi's Internal Classic*. According to this medical masterpiece, acupoints are the pores or channels for the infusion of *qi* and blood into the human body [5]. To be more accurately, the book also expounded the theory of Zangfu-meridian system, further explained that acupoints are the parts where *qi* and blood of the viscera and meridians are infused to the body surface. Some chapters like 'The Measurements of the Bones' and Discourse on Bone Hollows have described their concrete locations [5]^239,^ [12], just as bone hollow, muscle interspace and stirred pulse, respectively named as *gu kong*, *xi gu*, *mai dong* in Chinese. Acupoints are also called *qi* holes due to their powerful function of regulating *qi* and blood. *Miraculous Pivot*, the second part of *Huangdi's Internal Classic*, is to some extent a treatise specializing in acupoint and acupuncture. It elaborated acupoint selection and acupuncture therapy for exogenous diseases and internal injuries. From this point of view, acupoint being quite different from *marma*, appeared along with its standard medical methods in the beginning.

Obviously, although there are similar descriptions of morphology and function between *marma* and acupoint, their origins are quite discrepant. The former is derived from a battlefield culture, which consequently defined *marma* as an existence that needs careful protection and blessing as well as absolutely prohibition of injuries even the slightest one. Yet the latter is directly related to medical activities, encouraging people through moderate stimulations to avoid pain feelings and achieve a balanced state of maintaining health. Murthy, the translator and annotator of *S. samhita*, once concluded, “Though recognition of special spots on the body is common to both, the aim of approach of each one is thoroughly opposite of one another.” But overall, medical properties of these special points have been revealed in the origin process, and constantly influenced the evolution of respective medical behaviors.

## 3 General features

### 3.1 Location

**Fig. 1:**
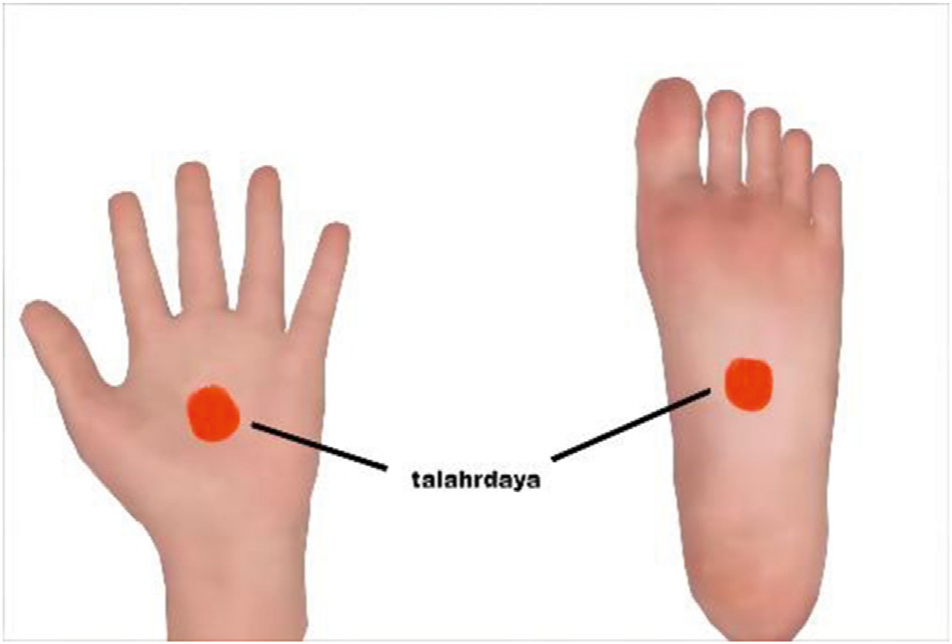
The location of *talahrdaya*.

**Fig. 2:**
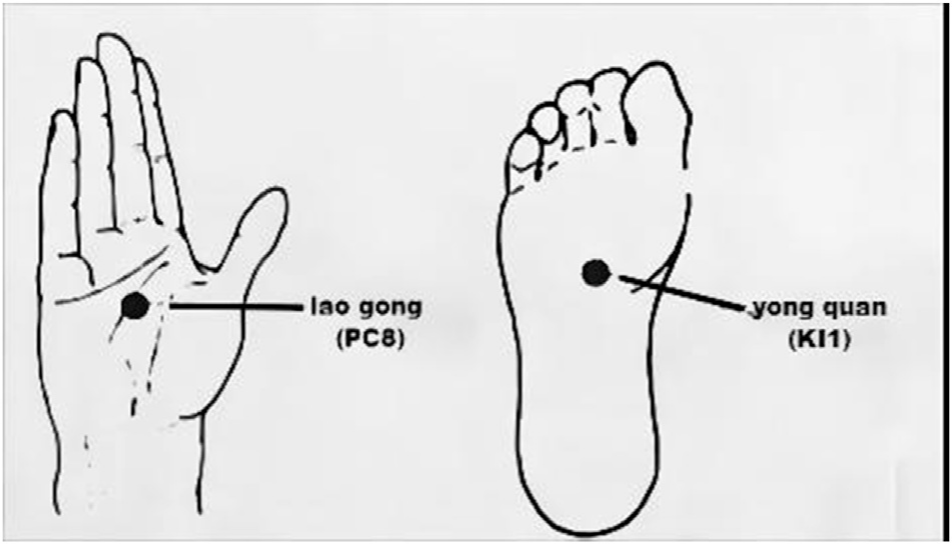
The location of *lao gong* (PC8) and *yong quan* (KI1).

The preceding part has mainly mentioned the location of *marma* and acupoint, just the junction of structural tissues. After a simple comparison, it is easy to recognize that the early site of them seems to be vaguely described with more overlaps. Take the vital point *talahrdaya* for example, when in the leg, it lies in the center of the sole and is in line with the middle toe; when in the hand, it lies in the center of the palm and is in line with the middle finger (see in Fig. 1). As Miraculous Pivot recorded [5], “qi of the kidneys exits through the *yong quan*. It is located on the sole of the feet” and “the *lao gong* is located in the lower joint of the middle finger”, locations of *yong quan* (KI1) and *lao gong* (PC8) were identified to highly coincide with *talahrdaya marma* (see in Fig. 2). Subsequently, knowledges of acupoint were carried forward by later generations in medical books due to their clinical practice value, and at the same time their positions continued to develop, gradually evolving to be accurate and specific in the later period. 'Great Compendium of Acupuncture and Moxibustion' in the Ming Dynasty documented body posture of receivers, which assisted to easily get the points. It defined that KI1 is the white boundary just trapped in the sole when the receiver sitting on heels curls the toes, and PC8 should be obtained by flexing the middle finger and ring finger in the palm [13]. Until modern times, descriptions of *marma* and acupoints location have been impacted by topographic anatomy and immediately developed into more accurate anatomical terminology [14,15]. Like the Ayurvedic book *'*Marma Points of Ayurveda*'*, the authors also introduced knowledge of modern anatomy to describe the locations of marma, and listed their corresponding acupoints in detail, for example, *talahrdaya marma* in the hand connected with PC8, *manibandha marma* with the *da ling* (PC7), *tarjani marma* with the *shang yang* (LI1) [16]. However, it should be reminded that even though these points tend to be more precise and standard, they can never be restrictedly positioned when used in clinical or actual operation, because for individuals, these special points can only be determined based on the principle of experiencing the flow of life energy.

### 3.2 Classification

There are several kinds of methods to classify these special points. In specific contents of physical distribution, *marma* in *S. samhita* could be divided based into five groups-the upper limb, the lower limb, the trunk, back, head and neck; but in *A-B* Classic of Acupuncture and Moxibustion, acupoints on the extremities are regarded as points of twelve meridians, the upper extremities being the three *yin* meridians and three *yang* meridians of hand, the lower extremities being the three *yin* meridians and three *yang* meridians of foot, while acupoints except extremities are divided into four groups-head and face region, neck, chest and abdomen, back region. Of element attributes, all 107 *marma* points could be classified by quality of fire, qualities of water and fire mixed together, quality of air, quality of water, qualities of fire and air; and in the system of acupoint, only the five transport points adequately match the five elements, while the others are not endowed with qualities of the five elements. Elemental attributes of these points are designed to further explain the reasons of diverse consequences after damage to these points, and by consequences there exists another classification, including rapid death, death after some time, death after removal of foreign bodies, malformation, and severe pain. Different from its focus on injury, the element attributes of five transport points are developed in order to better serve clinical practice and establish rules of treatment, such as mother-supplementing child-draining method. Also, to be clear, the five elements of Ayurveda are not the same as the five elements of TCM.

In addition, *marma* points focus on physical structure of human body, while acupoints are more integrated with medical theories to construct new classification methods. *S. Samhita* noted five varieties of names of *marma* under different anatomical classes, including *mamsa marma*, *sira marma*, *snayu marma*, *asthi marma* and *sandhi marma*, which respectively means muscular spot, venous spot, ligamental spot, bony spot and spot in joints. Later in *Astanga Hridaya*, *dhamani marma* representing arterial spot was added, but the total number was still 107 due to adjustment of the amount of each variety [3]. Such detailed descriptions and distinctions of physical structure suggested that practitioners at that time had a good command of anatomic knowledge. Murthy reminded that wars were frequent in ancient times in India, hence kings were taking surgeons to the battle field to treat the wounded. It can be said that the historical conditions provided a natural testing ground for improving knowledge of *marma*, which was once regarded as half of science of surgery in Ayurveda. In terms of acupoints, no structural classification has been made in spite of their different structural locations, just as “they are neither skin, flesh, sinews, or bones” documented in Miraculous Pivot [5], and the particularity lies in their intimate connection with meridians.

### 3.3 Other features

In more aspects, *marma* and acupoint reveal their respective characteristics as well as the similarity.

There is a large quantity gap between them. The number of *marma* has generally remained unchanged as 107 since the appearance of *S. Samhita*, in despite of existing the opinion of 108 *marma* points, yet roughly equal. While of acupoint, quantity of acupoint changed from 277 in Huangdi's Internal Classic, through 649 in *A-B* Classic of Acupuncture and Moxibustion and to 718 of modern national standard code (GB 12346-1990).

Just as acupoints connected to meridians, *marma* points are also contacted by internal energy channels named as *nadi*. They both carry normal vital energy in body's healthy state. In one study concerning history of acupuncture in India, authors have compared Ayurvedic *nadis* with TCM meridians, such as *Kuhu nadi* corresponding to Lung meridian [17].

## 4 Clinical application

In Murthy's perspective, only after clinical assessment of these special points in Ayurveda and TCM, can researchers perform the comparation and correlation between them. Undoubtedly, both of these body points have a long history of clinical application.

Clinical performance of *marma* has gone through two phases. As mentioned above, *marma* is a type of medical content derived from battlefield culture, and people once believed any stimulation towards these fatal points should be refused. Early clinical applications about *marma* placed emphasis on pathological knowledges. In the chapter 6 of *sharira sthana* in *S. Samhita*, hazards of injury and effects of injury were listed, revealing that consequences would be intractable and lingering, and patients may suffer dull pain, fatigue, emaciation, dizziness and many other symptoms [2]. Meanwhile, very few treatments were recorded. If *marma* points suffer extracorporeal injuries, the adjacent joint of the wounded should be amputated quickly in order to contract the blood vessels and stop bleeding [2]. Also, chapter 2 of *chikitsa sthana* described the person who has been hit on fatal spots, just same as an emaciated person, should be required to lie for some days in a tub filled with oil and given food along with meat soup [18]. These several scattered treatments in the book indicated its immature therapeutic rule focused on *marma* when they were more connected to traumatic injuries such as fractures and cut wounds. In the second stage, *marma*'s function of being a healing point emerged with relevant treatments. Known as a type of Ayurvedic therapy, *marma* therapy was actually a vein of medicine promoted by Ayurvedic doctors and practitioners of local martial arts in South India. That was when the martial art Kalaripayattu flourished and combined with science of *marma* points , that practitioners of Kalaripayattu adopted knowledges of body points as both a life protection and a health promoting technique, with a primitive application rule that a strong blow can cause injury while a mild touch can cause healing. It followed that the common perception on these points remained from wartime slowly changed, and *marma* points were no longer considered to be banned from stimulation. With the notion of mild touch, physicians developed treatments such as massage, application of herbal paste and medicinal oil and other gentle measures [[19], [20], [21]]. Under its influence, *marma* therapy was recognized as a kind of natural, non-invasive, durable method. Moreover, clinical data regarding efficacy of this therapy also showed beneficial outcomes, such as blood pressure normalizing effect and stroke rehabilitation [22,23]. Researchers also made reviews of *marma* therapy, and concluded that it assisted in diseases of single organ, systemic diseases involving multiple organs as well as acting as a response to many ailments that affect modern society [24,25]. In 2021, the authoritative book Marma Chikitsa-Basic Tenets in Ayurveda and Therapeutic Approaches published, introducing this time-honored and brilliant treatment in a very practical and comprehensible manner. Another application lies in its integration of yogic system, the posture of which affects the energy of *marma* in limbs, joints and spine, and during deep mediation *marma* are viewed as energy center in the whole practice [21]. Between the breath, the trainer becomes more sensitive to the flow of life energy and can master the skill to energize or clear *marma* points. That means yoga exercises through an interaction with *marma* points can also be conducted to achieve the state of health. In this way, a combination of medical usage and yoga focused on *marma* points may lead to a new direction.

Compared with step-by-step growth of *marma* points, acupoints seemed to be regarded as a one-step process. Acupoint therapies predominated by acupuncture have been on the history stage since the concept of acupoint took shape. Also acupuncture technique has been widely acknowledged to be responsible for the concentrated expression of acupoint knowledges. With assistance from tools applied to these points, ranged from the stone-needle of distant past to later nine classical needles and finally to disposable acupuncture needle nowadays, acupuncture operation has always been deeply and continuously bound to acupoints. During the long-term practice, acupuncturists discovered needling sensation, which resembled the phenomenon of meridian feeling transmission, and they assumed that only after a state of obtaining *qi*, that is, the patient gets feeling like soreness, numbness, heaviness, distention in the process of being pricked, can the healing effect be given full play. Another talented discovery consisted in various needling methods, including scattered needling, point-pricking, piercing, encircling needling and so on, all of which were put forward specifically because various methods could generate different effects for healing, while wrong selection of needling methods could even aggravate diseases. As for the selection of acupoints, doctors also found specific relationship between two or more acupoints during clinical experience and document research [26,27], and verify regulations like treating upper body disease through the lower body or he-mu point combination [28]. There being of course more medical applications in addition to acupuncture techniques, acupoint itself has played a significant role in the diagnosis, prevention and prognosis of diseases, and developed varieties of operations on it, for example, massage, acupressure, moxibustion, ointment [29,30]. And these years, a great number of relevant clinical studies were carried out, some of which even attracted worldwide attention [31,32]. To conclude, acupoints are related to medical experience from stem to stern, and for a long time closer than *marma* points in clinical application.

## 5 Discussion

Ayurveda and TCM are precious treasures of tradition medicine to mankind, and are still the most commonly used complementary and alternative medicine today. In a world of diverse diseases and increasing global health pressures, people are re-examining traditional medicine in an attempt to obtain the secrets of health recovery, health sustainability and life enhancement.

In this paper, we probed into some differences between *marma* points and acupoints. It is incontrovertible that they both belong to a category of human physical form, with the same phenomenon of “describing what people actually see and imagine together”. The energy and *qi* are invisible, but they are contained in the medical philosophy theories constructed by ancients, and once being connected with the objective structure of human body, their power functional concepts tend to be conceived, thus forming an inherent unify of structure and function. Maintenance of health results from proper performance of these structures and functions of the whole body.

As mentioned above, ancient Indians stimulated *marma* points to defeat enemies in wars, while ancient Chinese used acupoints to heal the patients. A few exceptions to this rule should be also noted. In the system of *marma*, *S. Samhita* once devoted a chapter named “phlebotomy”, which contained some descriptions about blooding-letting on these vital points [2]. For the instance of *ksipra marma*, a point located between the big toe and the next toe, can be cut for bleeding in foot diseases such as gout and sprained ankle. With regard to acupoints, there was a notable chapter named “acupuncture taboo” in A-B Classic of Acupuncture and Moxibustion [33]. It pointed out that some acupoints can't be stabbed at all, such as *ru zhong* (ST17) and *jiu wei* (RN15), and also some points are forbidden from deep insertion, such as *shang guan* (GB3) and *ren ying* (ST9).

Another problem we would like to argue is the relation of origin. Investigations have compared them based on a certain degree of similarity, and there once emerged a comment that modern acupuncture is rooted in *marma* therapy with the reason that practitioners of Kalaripayattu applied bamboo sticks on specific body points in order to treat as a long-standing tradition [34]. This point needs to be clarified. In China, acupuncture technique has never failed to be handed down from past generations, and at the beginning of the formation of acupoints, acupuncture theory and practice have been quite developed, so there is no doubt that modern acupuncture technology derived from its ancient tradition. It was also said that Ayurveda had a needle pricking treatment named *Suchi Veda*, but this practice did not flourish as its herbal medicinal treatment and even remained unrecorded throughout the Christian era and Middle Ages [17], probably due to people's fear for injuries of *marma* points. In the first century AD, Buddhism of ancient India was once introduced to China, although their philosophies and practices exchanged a lot and may led to mutual references for these special body points, the origin of acupuncture was still much earlier than cultural exchanges.

Finally, the effects and benefits of *marma* and acupoint should be emphasized and encouraged, and these vital points both make outstanding contributions to the health of all mankind since ancient times. Through the exploration of *marma* and acupoints, we sense more their significance, and realize that Ayurveda or TCM is not only being a medical system, but represents a healthy lifestyle that requires everyone to pay more attention to our body and mind.

In our view, both *marma* and acupoints pertain to the products of two cultural backgrounds which represent the early and independent body views of the two civilizations, and are more of two varieties of similar discoveries on special parts of the human body. From the descriptions of origin, general features and clinical application, we can realize that these points possess quite distinct cultural characteristics. Therefore, it would be indiscreet to evaluate both advantages and disadvantages without regard to cultural roots. Even though, the collision and comparison just show that there lies radical interconnectedness between the two medical systems: these two follow the holistic view and perceive the secret of life from the universe and nature. From the perspective of holistic integrative medicine, such comparative study can lay the foundation for the integration of medical theory and medical experience of traditional medicine. And after modifying and adjusting according to the reality of society, environment and psychology, a new medical system more suitable for human health could be worth expecting [35]. Cross-region comparison between traditional medicine assisted to achieve the state of cooperation and innovation. Moreover, tradition medicine cannot be explored by just focusing on technological improvement, because they not only carry the medical needs in different periods and regions, but also reflect people's cognitive level, as well as their attitudes towards the nature of the universe.

## Author contribution

Wu Tong: conceptualization, data curation, resources, writing and editing. Wang Xing-yi: supervision, validation, review & editing.

## Source of funding

Philosophy and Social Science Foundation of China (19VJX165).

## Declaration of competing interest

None declared.
